# Flexible Intumescent Roll-Form Fire Protection for Enhancing the Fire Resistance Ratings of Building Structures

**DOI:** 10.3390/polym18141736

**Published:** 2026-07-15

**Authors:** Marina Gravit, Vasily Prusakov, Olga Zybina, Muhammad Mudassar Chishti, Irina Kotlyarskaya, Maxim Sychov

**Affiliations:** 1 Civil Engineering Institute, Peter the Great Saint-Petersburg Polytechnic University, Polytechnicheskaya, 29 B, 195251 Saint-Petersburg, Russia; ozakata@mail.ru (O.Z.); chishti.m@edu.spbstu.ru (M.M.C.); 2 LLC “RPC PROMIZOL” St. Barclay 6c3, Office 7.05, 121087 Moscow, Russia; info@tdpromizol.com; 3 School of Advanced Engineering Studies of Digital Engineering, Peter the Great Saint-Petersburg Polytechnic University, Polytechnicheskaya, 29 B, 195251 Saint-Petersburg, Russia; iravassilek@mail.ru; 4 I.V. Grebenshikov Institute of Silicate Chemistry, 199034 Saint-Petersburg, Russia; msychov@mail.ru

**Keywords:** building structures, fire resistance, intumescent coatings, fire protection, swelling materials, intumescent flexible coatings

## Abstract

Intumescent coatings are widely used to enhance the fire resistance of structural steel. In contrast to traditional fire protection methods, this novel flexible intumescent protection offers several key advantages: universal compatibility with other coatings (via non-contact wrapping), resistance to extreme temperatures (−60 °C to +90 °C), all-weather usability, and suitability for light-gauge cold-formed thin-walled steel structures. This paper describes the development and investigation of these fire-protective, flexible intumescent coatings based on eco-friendly binders (silicone polymers and acrylic resins) with varying intercalated graphite (IG) content from 0% to 40%. An IG content of 25–40% enables a steel I-section with a section factor of 294 mm^−1^ to reach its limit state at 44 min (compared to 15 min for unprotected steel). Fire tests on steel beams with a section factor of 172 mm^−1^ demonstrated that samples reached the deflection limit state at the 64th and 66th minutes, respectively. Thermogravimetric analysis (TGA) was used to determine the temperature ranges for the thermal decomposition and expansion of the IG. Mechanical property studies revealed the influence of IG on the elastic modulus and tensile strength. Accelerated climatic testing in moderately cold conditions and salt spray chamber tests confirmed that the intumescent roll coating has no negative impact on the steel substrates.

## 1. Introduction

Fire protection is a set of technological and engineering solutions aimed at increasing the fire resistance of building structures and materials, reducing fire hazards, and ensuring the safe evacuation of people from buildings during a fire. Steel structures possess high strength at ambient temperatures but significantly lose their load-bearing capacity when heated above 500 °C [1], necessitating the use of specialized fire-protective materials. Methods for fire protection of steel structures are classified by the type of materials used and their application method. A long-established classification in [2] categorizes fire protection into two types: “wet” and “dry.” Wet methods involve the application of liquid-phase compositions (paints, varnishes, mastics) via spraying, brushing, or rolling, ensuring high adhesion to the protected surface. The “dry” method of fire protection for steel structures includes fireproof insulation in the form of boards or prefabricated components.

**Figure 1:**
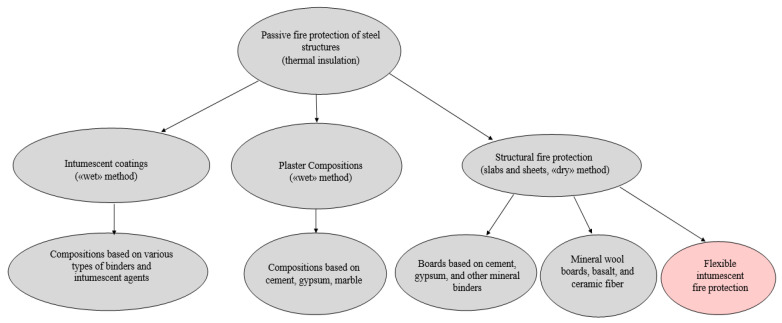
Classification of fire protection methods for steel structures.

A schematic of the full classification of fire protection methods is presented in Figure 1.

Intumescent coatings have become widely used, as they form a thermal insulating char layer when heated, which slows the heating of the metal [3]. The application technology for fire-protective coatings includes surface preparation (removal of contaminants and rust), application of a primer layer (to improve adhesion), and the direct application of the intumescent compound either manually or mechanically (by spraying). Another important method for protecting metal structures is structural fire protection in the form of boards and roll materials made from mineral fiber, vermiculite, or other non-combustible materials. These boards are mechanically fastened to the steel structures, providing reliable thermal insulation.

The advantages of fire protection using intumescent materials lie in their ease of application and high surface quality. The rapid growth in the use of intumescent materials in urban environments and industrial clusters is due to their minimal impact on the architectural aesthetics of exposed steel structures, as well as their low weight and flexibility for both on-site and off-site applications [4,5]. The industry focused on the development and application of intumescent fire-protective coatings is constantly evolving. In [6], the use of various fibers as hybrid reinforcement to enhance the mechanical strength of the coating was investigated, achieving the following results: a 15.7-fold increase in char expansion, a 22.8-fold reduction in weight loss, and a 37-fold improvement in charring performance under load. The introduction of nanosilica promotes the formation of a more compact char layer and effectively reduces heat and smoke release, thereby enhancing the fire-protective and smoke-suppressive properties of the coatings [7]. The coating formulated with 3 wt.% 300-mesh expanded vermiculite exhibited excellent fire resistance, with a comparative expansion of 30.43 times [8].

Study [9] proposed epoxy-based coatings incorporating plant-derived compounds (ginger powder and coffee husks) as a potential replacement for the carbon source in the intumescent system. Thermogravimetric analysis and fire resistance tests demonstrated the potential of these plant-based compounds as carbon sources, as they significantly lowered the temperature of the metal substrate. Furthermore, a research group [10] developed an organic–inorganic hybrid intumescent fire-protective coating containing industrial waste fly ash to enhance fire resistance and smoke suppression. Fly ash improves thermal insulation and strengthens the char surface. Finally, an intumescent coating based on a silicone-acrylic emulsion reinforced with palygorskite showed improved fire-protective and thermal insulation properties, as well as increased water resistance [11].

The authors in [12] presented an innovative solution in the field of passive fire protection for building structures: the development of a flexible, roll-form intumescent fire-protection system. This material combines the flexibility and ease of installation of rolled products with the thermal insulation properties of intumescent coatings and can be classified as a separate group (Figure 1). The base of this flexible roll-form protection is a fiber-reinforced polymer matrix (fiberglass or carbon fiber), integrated with intumescent additives—typically based on graphite and phosphorus-containing compounds. When exposed to high temperatures (approximately 200–300 °C), these additives decompose to form a porous, heat-insulating char layer that expands significantly in volume, effectively slowing heat transfer to the protected structure. Previously, a similar mesh was developed for cable structures using nitrile-butadiene rubbers.

The mesh (carbon or fiberglass) as a reinforcing matrix for fire-resistant epoxy and polyurethane-epoxy coatings is often used in composite materials [13,14,15,16].

**Figure 2:**
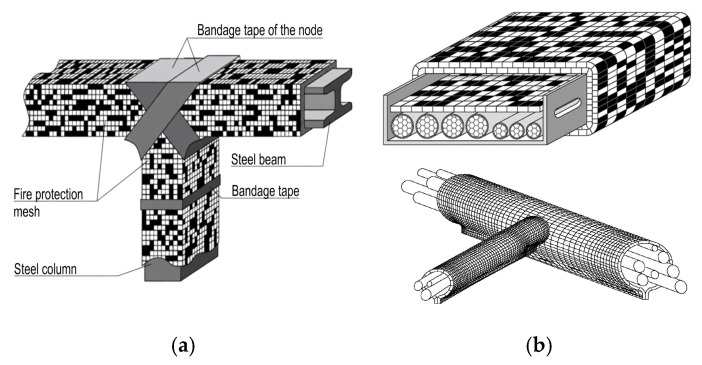
Installation diagram of the mesh on a steel structure: (**a**) wrapping a fire-protective mesh around a cable laid in a tray; (**b**) contact at the interface/connection point.

The detailed composition of the flexible rolled fire-protective material is described in the author’s patent [17]. Typical installation diagrams for the fire-protective mesh on steel structures, cable penetrations, and cable bundles are shown in Figure 2.

Flexible roll-form intumescent fire protection surpasses traditional methods due to its universal compatibility with existing coatings via non-contact installation; resistance to extreme temperatures (from −60 °C to +90 °C) and adverse environmental conditions (humidity, precipitation, and wind); and suitability for light-gauge cold-formed steel (LGCF) structures, thanks to its low weight and compactness. Additionally, it offers the possibility of manufacturing in various colors to match corporate branding. This fire protection system integrates structural, chemical (intumescent), and engineering solutions (Figure 1).

Similar fire protection has been established for forestry applications to protect trees from wildfires [18]. Patent [19] describes a mesh featuring an intumescent coating with expandable graphite and silica; the mesh comprises 35 to 56 wt.% acrylic copolymer, 3 to 21 wt.% ammonium polyphosphate, 3 to 21 wt.% expandable graphite, and less than 1 wt.% silica. One embodiment comprises a flexible grid with openings and an intumescent coating. This system was tested and found to comply with California building codes for soffits, which require that the assembly prevent embers and flames from passing through. The coating consists of expandable graphite, ammonium polyphosphate, and an acrylic copolymer carrier. However, the authors do not provide precise data regarding the fire protection efficiency of this coating.

Despite the many advantages of intumescent fire protection (as paints and varnishes), its behavior during fires has not been fully investigated. Scientific studies [5,20,21] confirm that such fire protection can behave inconsistently during a fire; the effectiveness of intumescent coatings and their thermophysical properties are significantly influenced by heating rates and environmental conditions. In Italy, according to [5], the design and assessment of steel elements protected with intumescent coatings are prohibited, as their performance under natural fire curves remains unknown in terms of both thermal and mechanical properties (e.g., adhesion). In Russia, it is prohibited to use intumescent coatings for steel structures with an effective metal thickness less than that specified in the regulatory document [22]. Over time, environmental exposure causes intumescent coatings, like most materials, to undergo aging, which can diminish their durability and thermal insulation properties. Established durability assessment procedures in Europe assume a service life of at least 10 years; for longer periods, additional evidence may be required [23].

In addition to LLC “RPC PROMIZOL” (Russia), there are several major manufacturers of fire-retardant intumescent mesh for metal structures: Hapuflam GmbH (Germany), Flamro (Germany), Genics Inc. (Canada), and Osmose Utilities Services (USA). These companies’ brochures claim that the fire-retardant effectiveness of their rolled fire-retardant compositions ranges from 15 min (one layer) to 60 min (two layers). However, they do not provide data on structural loads, specimen dimensions, the approximate chemical composition of the fire-retardant material, or the manufacturing technology. Based on the analysis of scientific literature, the primary challenges facing the scientific community in the field of intumescent fire protection include:

- Developing more environmentally friendly fire-protective composite materials with intumescent components;
- Expanding the database of large-scale experiments and diversifying research methodologies;
- Improving the modeling accuracy of the complex intumescence process;
- Enhancing the adhesion and cohesion of intumescent materials on various substrates, particularly steel;
- Predicting the long-term durability of fire protection for steel structures protected by intumescent materials.

In this study, a fire-protective roll-form coating using intumescent materials with more environmentally benign binders than those previously proposed by the authors in [12,17] was developed. In contrast, the researchers used nitrile butadiene rubbers dissolved in mixed solvents (ethyl alcohol and white spirit). In this case, acrylic (water-based) and liquid silicone rubbers (solvent-free) are used, which are more environmentally friendly than nitrile rubber coatings. Additionally, small-scale and large-scale fire tests, as well as climatic and mechanical testing, were conducted.

The aim of this research is to develop an intumescent roll-form fire protection system based on silicone and acrylic polymers with various combinations of intercalated graphite. The objectives of the study are:

- To identify formulations with the highest char residue, mechanical strength, and fire-protective efficiency;
- To investigate the resistance of these fire-protective coatings to climatic factors;
- To conduct experiments on steel samples with intumescent roll-form fire protection to achieve optimal fire-protective performance.

## 2. Materials and Methods

### 2.1. Material

Six variants of structural, flexible intumescent fire-protective coatings were developed based on silicone and acrylic binders. Coatings based on organosilicon and acrylic binders are known for their high thermal and oxidative stability, serving as reliable fire-protective materials. These coatings can expand due to the introduction of blowing agents, such as expandable graphite, organic compounds, and particles of hydrated alkali silicates [24,25].

The proposed fire-protective coatings consist of an elastic polymer composition based on silicone and acrylic binders, intercalated graphite, and other water-insoluble components, applied to a reinforcing mesh made from glass or carbon fiber composites.

**Figure 3:**
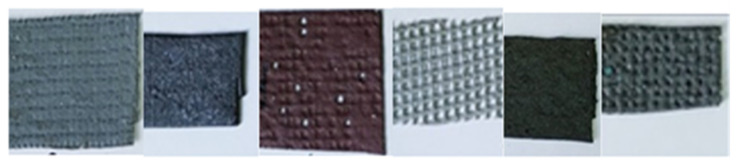
Photographs of the samples of the flexible intumescent fire-protective coatings.

The samples were produced in various color combinations by adding pigments, with variability in mesh aperture size and density depending on the applied composition and the type of reinforcing mesh. Samples No. 1–6 were prepared as sheet materials, which can also be manufactured in rolls (Figure 3).

In the fire protection systems considered in this article, the main expansion role will be played by expanded graphite (the system does not contain the triad “ammonium polyphosphate, melamine and pentaerythritol”). As is known from the literature [12,17,19], the amount of intercalated graphite in fire protection meshes can reach 21%. In our study, we are considering the thick (1.3–1.5 mm) composite material, where the amount of graphite can be increased to the limit values; therefore, the graphite content varied from 0% to 40%.

The samples differ from each other as follows:

- Sample 1: Based on an acrylic binder with an intercalated graphite (IG) content of 5%;
- Sample 2: Based on a silicone binder with an IG content of 25%;
- Sample 3: Based on a silicone binder with an IG content of 40%;
- Sample 4: Based on a silicone binder with an IG content of 0%;
- Sample 5: Based on an acrylic binder with an IG content of 40%;
- Sample 6: Based on an acrylic binder with an IG content of 25%.

**Figure 4:**
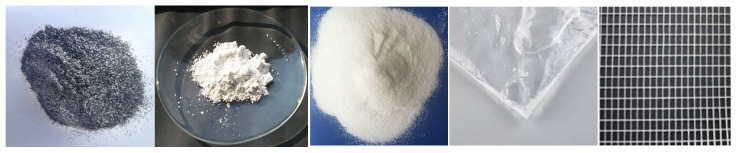
Graphite, antimony pentoxide, aluminum hydroxide, silicone rubber, and glass mesh (from left to right).

The formulations also contain aluminum hydroxide (20–30%), antimony pentoxide (3–7%), and titanium dioxide (5–10%). Photographs of the components are presented in Figure 4.

The properties of the EG-250 grade heat-expandable oxidized graphite (PJSC “Pigment”, Tambov, Russia) are summarized in Table 1.

The properties of aluminum hydroxide (manufactured by Chimpek, Moscow, Russia) are detailed in [26]. The properties of the silicone rubber are provided in [27]. The initial glass mesh had a thickness of 1.05–1.10 mm and an areal density of 80 ± 10 g/m^2^. The dispersion consists of an aqueous styrene-acrylic copolymer emulsion.

Table 2 presents the properties of the aqueous acrylic and silicone dispersions, which are liquids free of visible impurities. SKTN siloxane rubbers are low-molecular-weight dimethylsiloxane liquids, stabilized with active silica and cured using room-temperature vulcanizing (RTV) catalysts.

### 2.2. Manufacturing Technology

**Figure 5:**
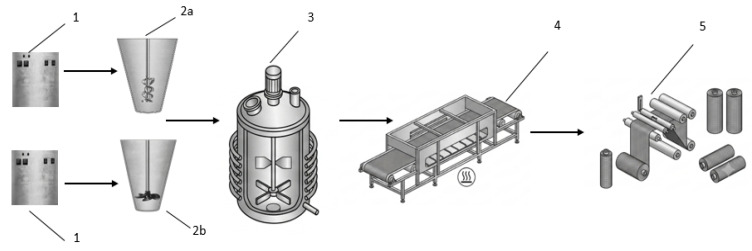
Schematic diagram of the preparation of fire-retardant rolled mesh using the spinneret spreading method: 1—controllers; 2a—dry material dispenser; 2b—liquid component dispenser; 3—dissolver for mixing components; 4—unit for feeding the fire-retardant composition through spinnerets, leveling, and drying; 5—cutting section.

There are several technologies for preparing rolled mesh with an intumescent fire-retardant coating: dipping (immersion) of the mesh into the finished composition [19], compression molding, or the method of spreading from spinnerets (dies) and leveling. This latter method is the one utilized by LLC “RPC PROMIZOL” (Moscow, Russia). The approximate manufacturing process is illustrated in Figure 5. Dry components are added to a container containing dispersions (liquid components) and mixed in a ball mill in accordance with the technological regulations of LLC “RPC PROMIZOL” until the required degree of grinding is achieved. Subsequently, the intumescent composition is applied to the fiberglass fabric via spinnerets; the material then passes through a drying chamber (with a temperature of +50–60 °C). Finally, the required number of sheets is cut using knives and wound into rolls.

### 2.3. Experimental Methods

#### 2.3.1. Determination of the Maximum Expansion Ratio

A SNOL-type muffle furnace was employed to determine the expansion ratio of the samples. The furnace operates by heating the muffle via surrounding heating elements, with the internal temperature monitored by a thermocouple and managed by a dedicated controller.

**Figure 6:**
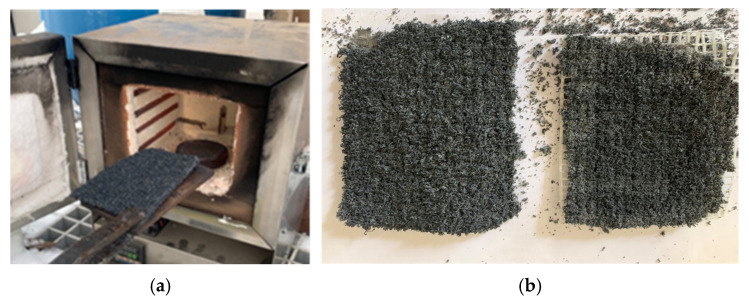
Determination of the swelling coefficient: (**a**) placement of the test specimen in the muffle furnace; (**b**) appearance of the expanded char layer for sample No. 5 after exposure to 500 °C and 600 °C for 30 min.

The samples were placed in a preheated muffle furnace at 600 °C and held for 5 min (Figure 6). The thickness of the resulting char layer was then measured, corresponding to the initial coating thickness [30,31]. The authors noted that the maximum height of the foam coke layer was also reached at 500 °C and decreased, similar to other types of coatings [32,33].

**Figure 7:**
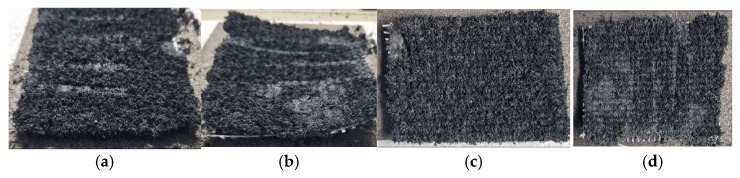
Appearance of the expanded char layer for different samples after furnace exposure for 30 min: (**a**) Sample No. 6 at 500 °C; (**b**) Sample No. 6 at 600 °C; (**c**) Sample No. 2 at 500 °C; (**d**) Sample No. 2 at 600 °C.

Previously, ref. [34] required the swelling coefficient to be 500 °C for 30 min, which turned out to be inappropriate for this study, since during this time the foam coke network elongates and competes in height (burns out) in measurement with other samples. As can be seen in Figure 6 and Figure 7, the difference in swelling coefficients at 500 °C is, on average, equal to the coefficients dependent on the temperature at 600 °C for 30 min. In December 2025, a change was made to the standard [34], which also sets 600 °C for 5 min and is consistent with standards [31]. In addition, the expansion coefficient of foam coke of various coatings in different temperature–time intervals requires separate study (expansion coefficients differ depending on the types of film formers, fillers, and types of foaming agents).

#### 2.3.2. Microscopic Examination

An Altami CM0745 stereomicroscope (LLC “Altami”, Saint Petersburg, Russia) [35] was used for microscopic examination. The instrument is designed for the observation of three-dimensional images in both reflected and transmitted light. Images were captured after the samples had undergone fire testing.

#### 2.3.3. Thermal Analysis

A synchronous thermal analyzer, the STA 6000 (PerkinElmer, Shelton, CT, USA) [36], was used for thermal analysis. This analyzer is an integrated system that combines the functions of a differential scanning calorimeter (DSC) and a high-sensitivity analytical balance. This configuration allows for simultaneous measurement of calorimetric values during various thermodynamic transitions, the determination of transition temperatures, and the continuous registration of mass changes within a single experiment on a single sample. The following experimental conditions were selected for the analysis: heating in an air atmosphere up to 1100 °C at a rate of 15 °C/min, followed by cooling at a rate of 30 °C/min.

#### 2.3.4. Small-Scale Fire Testing

All specimens were tested in a small-scale furnace in accordance with [37]. The furnace is constructed of masonry with internal dimensions of 790 mm (width) × 790 mm (length) × 965 mm (height) and is equipped with two diesel burners. The furnace temperature regime is monitored by two Type K thermocouples (TP-0395-06HA, PC “Marion”, St. Petersburg, Russia) with an insertion depth of 500 mm.

Test samples No. 1–6 were mounted on No. 20 I-beam profiles with a section factor of 294 mm^−1^ and a height of 750 mm. The temperature of the test specimen is monitored by three thermocouples: one in the middle section on the I-beam web and two on the inner surfaces of the I-beam flanges. The metal temperature is determined as the arithmetic mean of these three readings. Data are transmitted to a computer via an interface converter. The experiments are conducted under zero static load, providing four-sided thermal exposure until the I-beam profile reaches its limit state (a temperature of 500 °C).

#### 2.3.5. Study of Mechanical Properties (Tensile Modulus of Elasticity)

The mechanical strength of the fire-protective samples was tested according to [38]. The test specimen is subjected to tension along its primary longitudinal axis at a constant rate. During the test, the applied load and specimen elongation are continuously measured to determine the required mechanical indicators. The material types and test parameters are presented in Table 3.

Since the test material is anisotropic, at least five specimens are prepared, cut both longitudinally and transversely relative to the primary material direction.

The specimen is loaded at a jaw separation speed that ensures a specimen strain rate of (1.0 ± 0.5) % per minute. A graphical “Load–Deformation” plot is recorded during the test. The tensile modulus of elasticity, *E_p_*, is calculated using Formula (1):

(1) $$E_{p}=\frac{(F_{2}-F_{1})\cdot l_{0}}{A_{0}\cdot (\Delta l_{2}-\Delta l_{1})}$$

where

*F*_2_—the load corresponding to a relative elongation (strain) of 0.3%, N;

*F*_1_—the load corresponding to a relative elongation (strain) of 0.1%, N;

*l*_1_—the initial gauge length of the specimen, mm;

*A*_0_—the initial cross-sectional area of the specimen, mm^2^;

Δ*l*_2_—the elongation corresponding to load F_2_, mm;

Δ*l*_1_—the elongation corresponding to load F_1_, mm.

#### 2.3.6. Durability Assessment of Fire-Protective Coatings Under Climatic Exposure

Each type of fire-protective coating system utilizes a unique combination of materials with diverse physical and chemical properties. When fire-protective coatings are applied directly to steel, most manufacturers recommend using primers selected for compatibility with the specific coating while meeting corrosion protection and environmental requirements. The primer ensures adhesion to the substrate under standard service conditions, provides corrosion protection, and maintains the bonding of the char layer as it expands during a fire.

The methodology outlined in [31] is employed to investigate the impact of climatic factors on the fire-protected steel structures. The essence of this method involves conducting accelerated weathering tests on steel plate specimens with applied fire-protective coatings. This is followed by an assessment of the coatings’ resistance to climatic exposure and their ability to retain both fire-protective and anti-corrosive properties throughout their intended service life.

The specimen consists of a sheet steel plate measuring 600 × 600 × 5 mm, with the fire-protective coating applied to its obverse (front) side. The reverse side and the edges of the plates must be coated with a compatible paint or varnish material to protect these surfaces throughout the duration of the accelerated weathering tests. In this study, an epoxy primer (manufactured by “VMP”, Yekaterinburg, Russia) is utilized; this primer is widely used both within multi-layer anti-corrosive systems for concrete and metal surfaces and as a standalone protective coating.

To evaluate resistance to climatic factors, the specimens were placed in a temperature-humidity-cold environmental chamber (Model SM-70/100 TVKh, SPM Climate, Saint Petersburg, Russia). To assess resistance to neutral salt spray, the specimens were exposed to an atmosphere saturated with NaCl. The testing procedure was conducted in accordance with Method B [39].

During both tests, adhesion was assessed to ensure it was sufficient for the fire protection system to withstand mechanical impacts and protect the substrate from corrosion. Poor adhesion can significantly reduce the service life of fire protection materials, risking total failure under loads such as high-pressure water flow from a fire hose. Adhesion was evaluated using the cross-cut method. According to the aforementioned standards, the post-test adhesion of the coating must be rated no worse than Grade 3. The permissible degradation of properties during service for high-gloss and glossy coatings treated with a polishing compound is no worse than Grade 2; without polishing treatment, it is no worse than Grade 3.

#### 2.3.7. Large-Scale Testing

Testing of pilot steel beam samples for fire exposure to determine their fire resistance limit is performed in accordance with [40], provided that the furnace’s fire chamber creates the appropriate temperature regime.

For the large-scale fire tests, beams No. 30B1 measuring 3000 mm in length were used, equipped with fire protection mesh and threading (t_red_) of 3.4 and 4.3 mm; the applied loads were 68.67 and 81.63 kN, respectively. The beam samples for load-bearing tests are equipped with support devices (stops) for installation in the loading apparatus, ensuring pinned (fixed) and roller (movable) support at the ends, to ensure structural stability.

The sample is tested under three-sided thermal exposure with a static load applied using a two-point scheme in each third of the span length. The test result is taken as the time (in minutes) from the onset of thermal exposure up to the occurrence of the limiting state of the sample. For bending structures, the limit state is considered to occur if the deflection reaches L/20 or the deformation rate reaches L^2^/9000 h cm/min, where L is the span in cm; h is the design height of the cross-section in cm.

## 3. Results

### 3.1. Muffle Furnace Testing

Samples No. 1–6 were placed in a muffle furnace preheated to 600 °C and held for 5 min in accordance with [31]. After cooling to room temperature, the thicknesses of the char layers of samples No. 1–6 were measured; the resulting dimensions are presented in Table 4.

**Figure 8:**
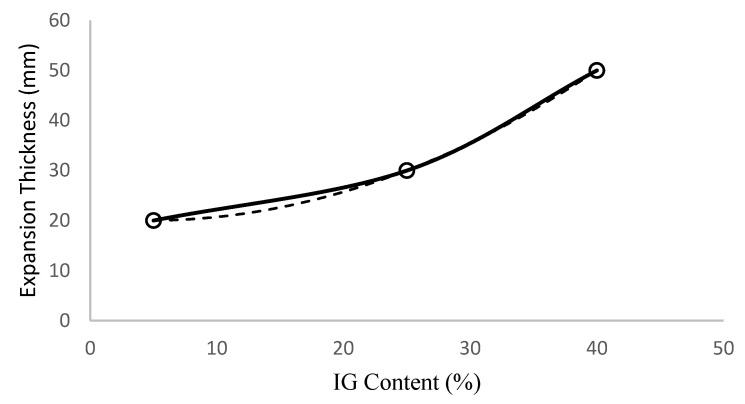
Dependence of char layer height on graphite content.

Figure 8 shows the graph of the char layer growth as a function of graphite concentration.

As shown in Figure 8, a directly proportional relationship is observed.

### 3.2. Microscopy

After heating in the muffle furnace, the char layer formed by the action of fire on samples No. 1–6 was photographed at standard resolution and under the scanning electron microscope (SEM) [35]. The results are presented in Table 5. Technical characteristics of the study (shown in the photographs in Table 5): SEM HV (High Voltage): 20.0 kV; SEM MAD (Magnification) (3.02 kx–6.62 kx); WD (average value): 15.28 mm; View field: 63.7–137 mm. Scale bar: 20 mm. Det: SE (the type of sensor that collected the signal. SE (Secondary Electrons)).

It can be seen that the graphite flakes have different shapes and thicknesses of approximately several tens of nanometers.

For all samples, the foam coke was sufficiently rigid and adhered firmly to the substrate. For sample № 4, the char layer was extremely brittle, making it impossible to obtain high-quality photographs. Thermal shock causes expansion of the intercalated graphite due to the evaporation of intercalite and water molecules, leading to extrusion of the graphite layers and the formation of a multilayer thermally expanded material in the form of “worms” that are highly porous and weakly bonded. The following types of porosity in the worms are distinguished: interparticle porosity—the porous space between curved worms and between irregularly oriented carbon plates. An ordered structure was observed, particularly in samples 2, 3, and 5. This structure predicts more stable properties in both mechanical characteristics and fire protection efficiency.

### 3.3. Thermogravimetric Analysis

**Figure 9:**
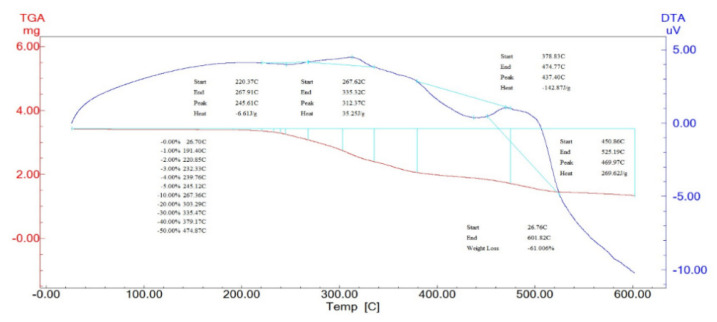
Acrylic binder with a 5% IG content in the mesh (Sample 1).

**Figure 10:**
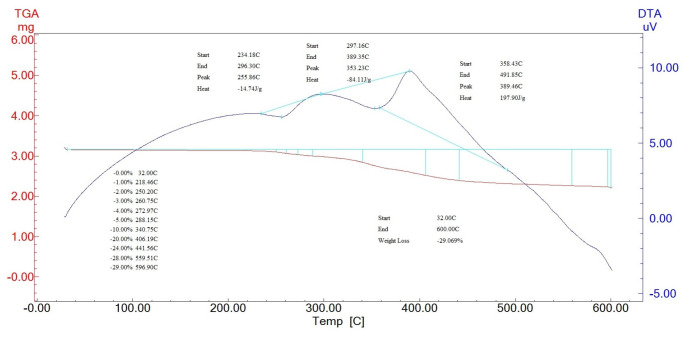
Silicone binder with a 25% IG content in the mesh (Sample 2).

**Figure 11:**
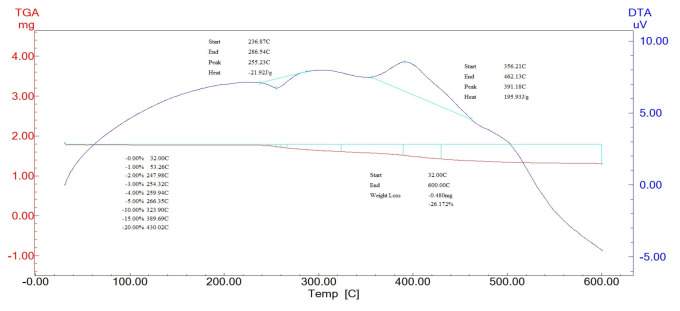
Silicone binder with a 40% IG content in the mesh (Sample 3).

**Figure 12:**
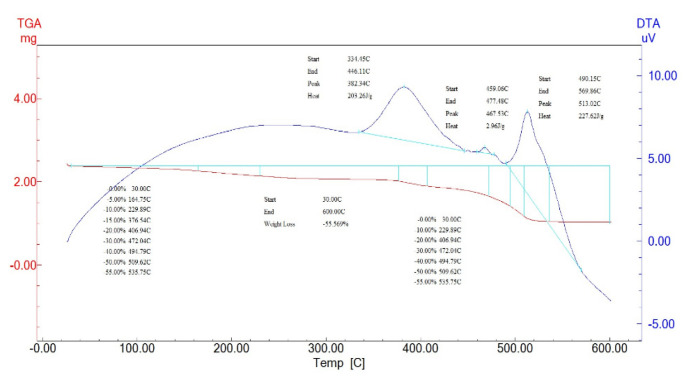
Silicone binder with a 0% IG content in the mesh (Sample 4).

**Figure 13:**
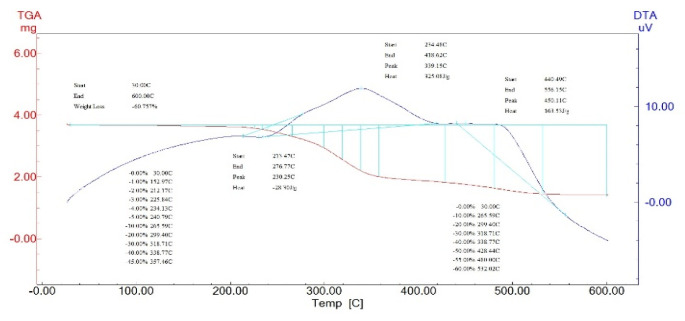
Acrylic binder with a 40% IG content in the mesh (Sample 5).

**Figure 14:**
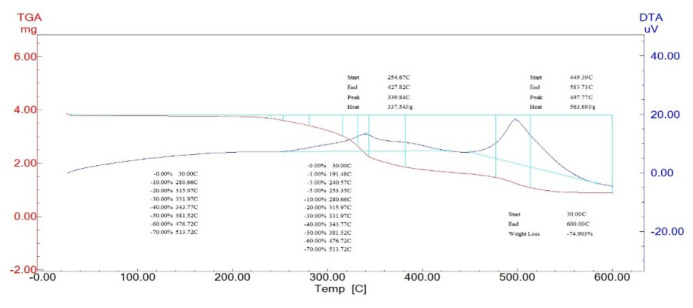
Acrylic binder with a 25% IG content in the mesh (Sample 6).

Thermal analysis was conducted on samples No. 1–6 to determine the onset of thermal decomposition and the expansion of intercalated graphite at various concentrations. The TGA curves for the samples are presented in Figure 9, Figure 10, Figure 11, Figure 12, Figure 13 and Figure 14.

Clearly, peaks of carbon framework burnout (400–600 °C) are observed, typical of intercalated graphite in an air atmosphere. The main peak of deintercalation and swelling occurs at 150–300 °C due to new gases (SO_2_, CO_2_, H_2_O, NO_x_), accounting for a difference of 10–30% of its mass. Table 6 presents the key parameters derived from the thermal curves for all samples.

For sample No. 3 (Figure 11), the onset temperature of decomposition with an increase of 1 °C was only 53.26 °C, which is inconsistent with other results for samples with silicone binders (S). However, if we look at the data further (−2.00%; −5.00%, etc.), the mass loss with increasing temperature shows quite similar values.

### 3.4. Small-Scale Fire Tests

During the experiment, the following parameters were recorded: the time taken for the I-beam profile to reach its critical state (temperature of 500 °C); the temperature dynamics in the furnace chamber in accordance with ISO 873 requirements (standard fire temperature conditions); visual characteristics of the fire protection coating’s reactions to fire (formation of an intumescent layer, carbonization, delamination, formation of cracks, emission of smoke and volatile pyrolysis products, etc.); and changes in the temperature of the metal in the test specimen.

**Figure 15:**
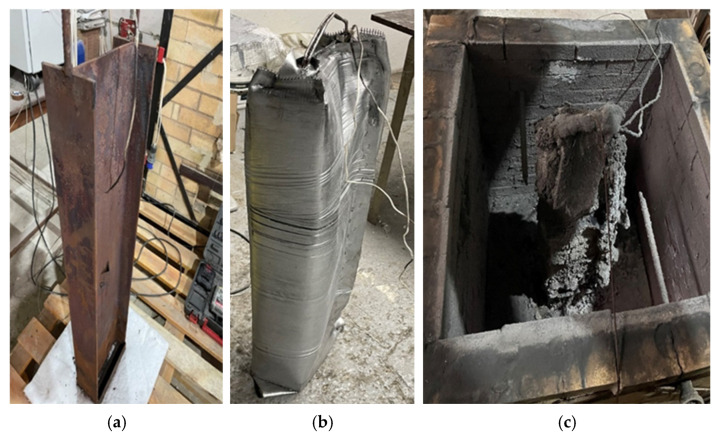
Beam specimen without fire protection (**a**), with fire protection (**b**), and after testing with fire protection (**c**).

Figure 15 shows an unprotected I-beam and a sample with an applied fire protection coating.

**Figure 16:**
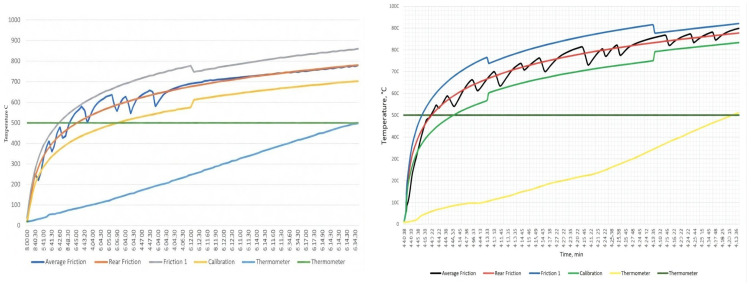
Temperature–time curves for mesh samples No. 1 and 2.

Figure 16 presents the temperature–time curves for mesh samples No. 1 and 2. The graphs display data from the furnace thermocouples and the thermocouples located on the unexposed surface. The values obtained during the small-scale fire tests are summarized in Table 7.

Table 7 summarizes the values obtained during the small-scale fire tests.

**Figure 17:**
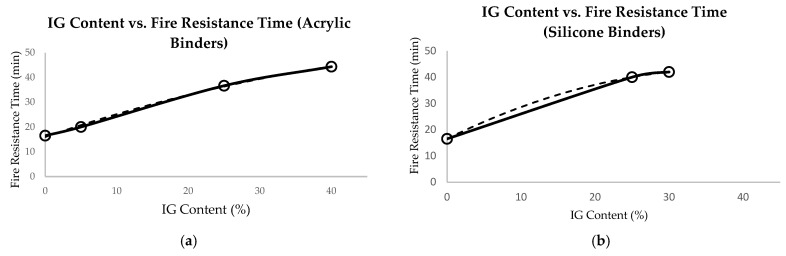
Graphs with acrylic binders (**a**) and silicone binders (**b**).

Based on the results of the tests, dependency graphs (or relationship plots) were constructed. Figure 17 illustrates the dependence of fire resistance time on the intercalated graphite (IG) content for materials formulated with acrylic (a) and silicone (b) binders.

### 3.5. Investigation of Mechanical Properties

**Figure 18:**
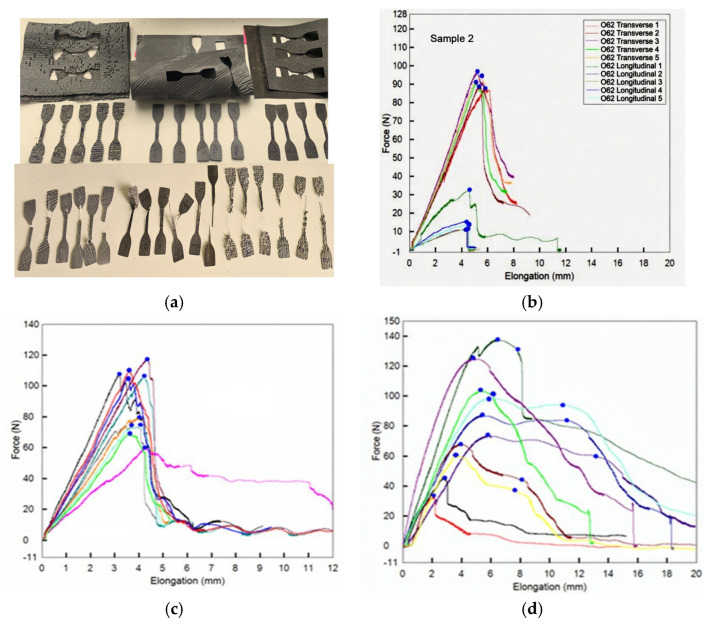
Investigation of material mechanical properties: (**a**) specimens for mechanical testing, (**b**) curves for sample No. 2, (**c**) curves for sample No. 3, (**d**) curves for sample No. 5.

As shown in Table 7, the introduction of intercalated graphite within the range of 5–40% clearly leads to an increase in the fire-protective efficiency of the composition. For the samples that demonstrated the highest fire-protective performance (Samples No. 2, 3, and 5), the elastic modulus and the loss of tensile strength were investigated (Figure 18). Table 8 summarizes the obtained mechanical property values.

### 3.6. Investigation of Resistance to Climatic Factors

**Figure 19:**
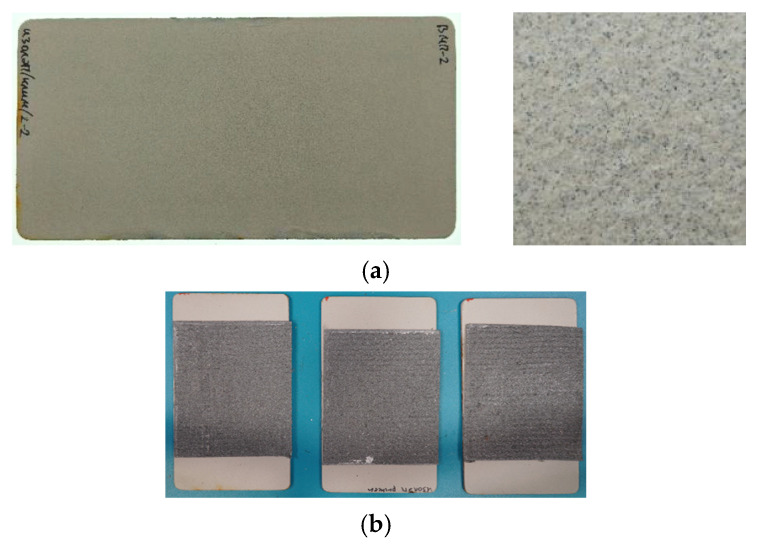
Appearance of the VMP-system after accelerated climatic testing: (**a**) without fire-protective overlays; (**b**) with fire-protective overlays.

No changes in the appearance of the primer coating were observed following the accelerated climatic tests. Figure 19b illustrates the appearance of the specimens after accelerated climatic testing with the flexible roll-form fire protection overlay (Sample No. 5), which demonstrated the best performance in the small-scale fire tests. The appearance of the VMP system surface beneath the overlays was indistinguishable from the surface areas without overlays (Figure 19a). No corrosion products were detected in the investigated section of the specimen.

**Figure 20:**
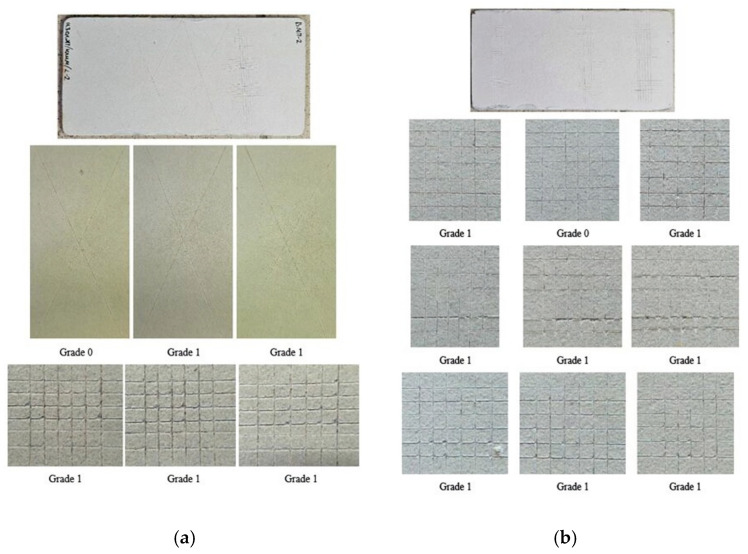
Appearance of the notched specimen after exposure to high humidity, temperature, and UV radiation: (**a**) without mesh; (**b**) with the fire-protective mesh.

To determine the adhesion of the fire-protective coatings, cross-cut and X-cut notches were made on the specimens. Figure 20 illustrates the appearance of the notched specimens after climatic testing, both with and without the fire-protective mesh on the primed plates.

**Figure 21:**
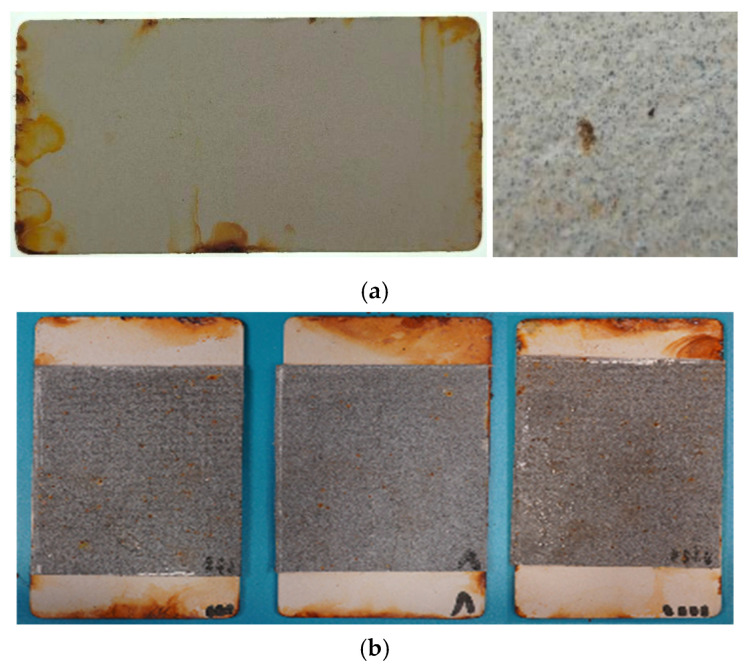
Appearance of the VMP system after salt spray chamber testing: (**a**) without fire-protective overlays; (**b**) with fire-protective overlays.

**Figure 22:**
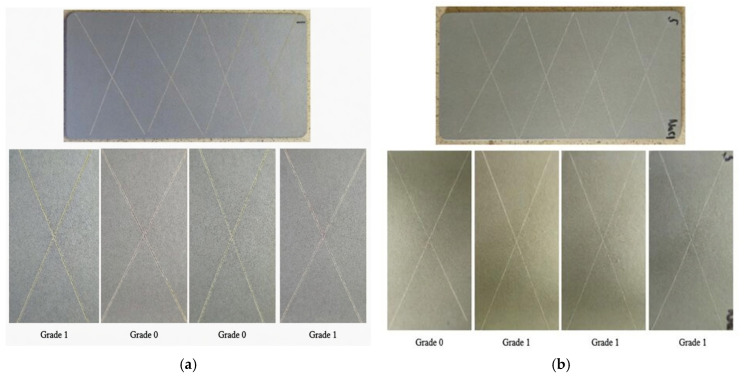
Appearance of notched primed specimens after salt spray exposure: (**a**) without mesh; (**b**) with the fire-protective mesh.

Following testing in the salt spray chamber, the formation of isolated corrosion products was observed on the surface of the VMP primer (Figure 21a). The primary corrosion products formed on the edges of the specimens. The test results for the VMP primer system with the fire-protective mesh were analogous to those of the system without the mesh overlay (Figure 21b). Figure 22 shows the appearance of notched specimens after exposure to salt spray.

The test results are systematically presented in Table 9. Based on the results of the accelerated weathering and salt spray chamber tests, the mesh did not affect the properties of the coatings.

### 3.7. Large-Scale Fire Testing

Installing firestop mesh does not require any special surface preparation of steel structures. To conserve firestop mesh, it is recommended to pre-cut standard wrapping assemblies and install them using these templates.

**Figure 23:**
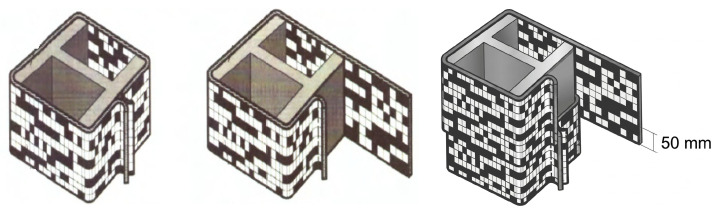
Method of laying fireproof mesh, including overlapping.

Mesh installation is typically performed as follows: wrap the mesh around the structure and secure it with staples using a tacker, as shown in Figure 23. When wrapping I-beam columns with firestop mesh, ensure that the mesh sheets overlap by at least 50 mm when joined.

**Figure 24:**
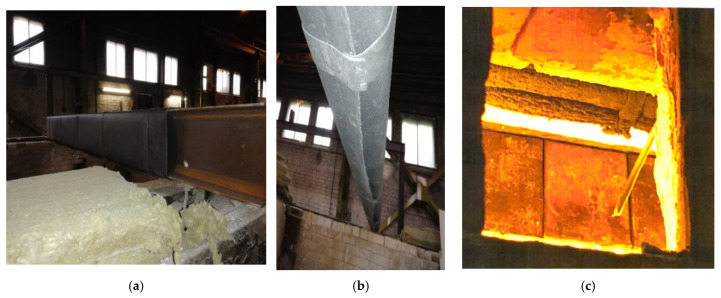
Conducting fire tests: (**a**) wrapping the mesh around the beam, (**b**) securing the mesh with staples; (**c**) conducting fire tests, and inspection through the inspection window at the 5^th^ minute of prototype testing.

**Figure 25:**
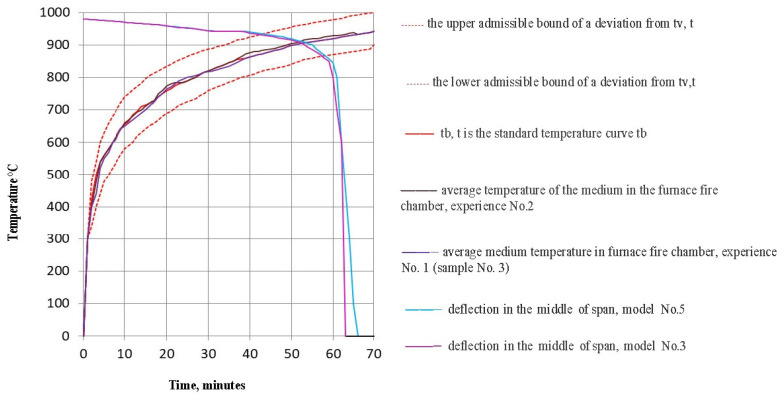
Temperature profiles in the furnace chamber and deflection curves for the fire-protective mesh specimens.

The best fire-protective performance in the small-scale fire tests was demonstrated by Sample No. 3 (silicone binder) and Sample No. 5 (acrylic binder). For the large-scale fire tests, No. 30B1 beams with the volumetric coefficient of metal being 294 mm^−1^ and 239 mm^−1^ (or reduced metal thickness of 3.4 mm and 4.3 mm [37]) were used, equipped with fire-protective mesh and subjected to loads of 68.67 kN and 81.63 kN, respectively (Figure 24 and Figure 25). In these tests, the areal density of the mesh was increased to 200 ± 15 g/m^2^, utilizing a composition with 100% mesh cell filling and an increased total thickness of 2.0–2.5.

Figure 24 illustrates the mesh installation and fire testing process, performed in accordance with standard testing protocols [40].

**Figure 26:**
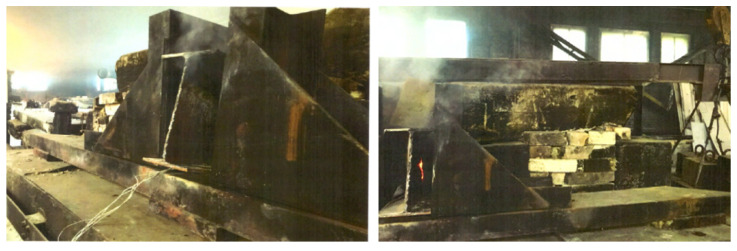
Specimens reaching the load-bearing limit state (R).

Thermal expansion begins at the 5–8 min mark, followed by the whitening of the expanded coating layer observed between 45 and 50 min. At the 64^th^ minute for Sample No. 3 and the 66^th^ minute for Sample No. 5, the specimens reached their limit state. This was defined by the deformation rate exceeding 0.33 cm/min and an ultimate deflection of over 200 mm, characterized by rapid deflection growth and the subsequent structural collapse of the test specimens (Figure 26).

## 4. Discussion

Muffle furnace and small-scale fire testing revealed that an optimal intercalated graphite (IG) content of 0–40% significantly extends the time required for an I-beam profile to reach its limit state (up to 44 min). This corresponds to Group 6 fire-protective efficiency according to regulatory requirements.

The authors studied compositions with higher graphite contents: 50% for samples with styrene-acrylic binders and 60% for samples with silicone binders, resulting in foam coke heights of 70 mm and 35 mm, respectively. This graphite concentration is critical, as higher graphite contents lead to brittleness, fragility, and cracking of the network, as well as to graphite loosening depending on the foam height. Therefore, the fire resistance and strength parameters of such coatings were not studied.

Thermogravimetric analysis (TGA) enabled the determination of the specific temperature ranges for the thermal decomposition and expansion of the IG, which is essential for understanding the operational mechanism of these fire-protective compositions. Studies of mechanical properties indicated that the inclusion of IG influences the elastic modulus and tensile strength; these factors must be accounted for when developing formulations that require an optimal balance of fire resistance and mechanical performance.

As is well known, silicones have an inorganic siloxane bond (Si-O), which exhibits high bond activity and ensures thermal stability over a wide temperature range. Acrylic polymers (based on acrylic or methacrylic acid esters) have an organic carbon backbone (C-C), which, at elevated temperatures or during aging, undergoes more extensive degradation and releases volatile components. The chemical nature of the polymers, as confirmed by the test results for the samples (Table 6), includes total mass loss values, clearly categorized by binder type:

- Silicone-based: Sample 2 (25% IG): 29.07%; Sample 3 (40% IG): 26.17%; Sample 4 (0% IG): 55.57%;
- Acrylic-based: Sample 1 (5% IG): 61.00%; Sample 5 (40% IG): 60.7%; Sample 6 (25% IG): 74.90%.

Based on the chemical composition of the film-forming substances, it can only be confirmed that Sample 5, containing the highest amount of graphite, swells the most (50 mm), but loses 60.7% of its weight. The silicone sample (No. 3) exhibits the same swelling (50 mm), but loses less weight—26.2%. These samples demonstrated fire protection efficiency (reaching 500 °C) for Sample 3–44.2 min, and for Sample 5–43.30 min.

Electron microscopy could not capture photographs of Sample 4 (without graphite in the silicone binder), as the foam-like char proved to be extremely brittle. The samples of the other compositions had a fairly regular structure, with the best being Sample 5 (40% graphite with acrylic dispersion), as well as Sample 2 and Sample 3 (silicone polymer composites with 25% and 40% graphite, respectively).

Large-scale fire tests of two No. 30B1 steel beams equipped with fire-protective mesh under loads of 68.67 kN and 81.63 kN yielded the following results: thermal expansion initiated at 5–8 min, with whitening of the expanded layer observed at 45–50 min. The flexible intumescent fire protection specimens that performed best in small-scale tests (Samples 3 and 5) reached their load-bearing limit state (R) at 64 and 66 min, respectively. Failure was characterized by a deformation rate exceeding 0.33 cm/min and an ultimate deflection of over 200 mm, resulting in rapid deflection growth and final structural collapse.

Based on the results of accelerated weathering and salt spray chamber tests, the flexible intumescent fire-protective coating did not adversely affect the properties, structural integrity, or durability of the protected substrate. Further research is required for this type of fire protection regarding the influence of fixation methods on structural strength and the potential for environmental degradation under specific operating conditions.

According to Russian regulations [22], intumescent paints are permitted for structures with a fire resistance rating of R60 or higher, provided the volumetric ratio of metal in the structure exceeds 172 mm^−1^. Fire-retardant intumescent mesh (not paint) is certified as a structural fireproofing material. This allows it to be used to protect steel structures with any fire resistance rating. It should be noted that fire resistance requirements for buildings vary across countries and even states (for example, New York City authorities adhere to strict fire resistance and building code requirements (R180-240) due to the high risk of development in these states and the history of fires in the United States. On the other hand, states such as Texas and Florida have more moderate requirements (R60-R180) due to climate conditions and typical low-rise buildings [41]).

## 5. Conclusions

Intumescent coatings are used to improve the fire resistance of structural steel. Unlike traditional fire protection methods, flexible intumescent fire protection—a polymer composition integrated into a non-combustible mesh base—offers the following advantages: universal compatibility with previously applied fire-protective or anti-corrosive coatings (via non-contact wrapping), a wide operating temperature range (–60 °C to +90 °C), all-weather usability, simplified inspection and repair, suitability for light-gauge steel framing (LGSF) due to its low weight and compactness, and the potential for mass production in various colors.

In this study, six variants of structural, flexible, intumescent fire-protective coatings were developed and evaluated. These coatings utilize eco-friendly silicone and acrylic binders (free from solvents and halogenated products) with varying intercalated graphite (IG) content.

The authors plan to conduct further research into the forms of gaseous components during combustion (to determine the toxicity of pyrolysis products). Also of interest is research into acrylic-silicone binders, which could lead to the development of heat-resistant silicone coatings with the lower cost and processability of acrylic-styrene binders. The addition of montmorillonite, carbon nanotubes, vermiculite, and other synergistic fillers can increase the structural strength of foam coke, reduce cracking, and enhance thermal insulation.

## Figures and Tables

**Table 1:** Properties of intercalated graphite.

Parameter	Carbon Content, %, Not Less Than	Ash Content, %, Not Less Than	Expansion Rate, at Least, cm^3^/g (1000 °C)	Particle Size (50 mesh), %, Not Less Than	Expansion Ratio, Not Less Than	Moisture, %, Not More Than
Value	95–96	5.0–4.8	250–260	90	150–155	1.0

**Table 2:** Properties of aqueous acrylic and silicone dispersions.

Dispersion Type/Parameter	Appearance	Non-Volatile Matter Content, %	Dynamic Viscosity, Pa·s *	Min. Film-Forming Temperature, °C	Frost Resistance (Cycles) at T = −(20 ± 2) °C, min	Transportation and Storage Temperature
Acrylic dispersion SB 305 [28]	Milky white	49 ± 1	0.7–1.5	16	4	No less than +5 °C and no more than +35 °C
Silicone dispersion [29]	Viscous, colorless, cloudy	98.0	1.8–2.5	+5 … +10	500–100	Not exceeding +30 °C, away from direct sunlight

Note * Measured at (23 ± 0.5) °C using a Brookfield RVDV-II + PRO viscometer (Middleborough, Massachusetts, USA, Spindle 2/20 rpm/23 °C), Pa·s.

**Table 3:** Test specimen characteristics.

Specimen Type	Parameter	Value, mm
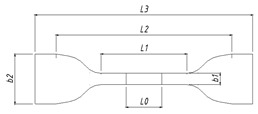	Gauge Length, L_0_	25 ± 1
	Length of narrow parallel-sided section (working section), L_1_	33 ± 1
	Distance between broad parallel-sided sections, L_2_	80 ± 5
	Overall Length, L_3_	>115
	Width of narrow section (working zone), b1	6 ± 0.4
	Width of grip section (head), b_2_	25 ± 1

**Table 4:** Thicknesses of intumescent layers after heating.

Sample No.	1	2	3	4	5	6
IG Content	5%	25%	40%	0%	40%	25%
Initial mesh thickness, mm	1.2	1.3	1.4	1.2	1.5	1.4
Intumescent (char) layer thickness *	20 mm	30 mm	50 mm	20 mm	50 mm	30 mm

* Mean value.

**Table 5:** Photographs of the char layer at standard resolution and under the SEM.

Sample No	Char (Foam-Coke) Photograph	SEM Cross-Sections of the Char (Foam-Coke)	Char (Foam-Coke) Morphology
1	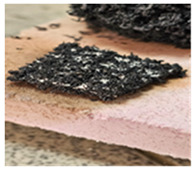	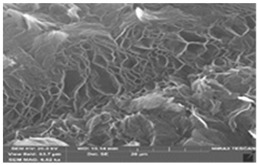	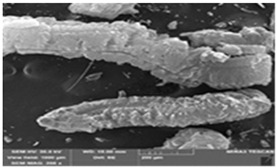
2	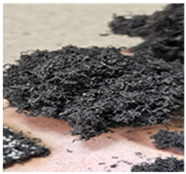	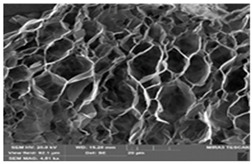	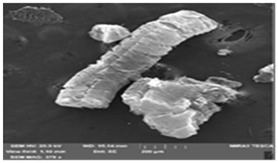
3	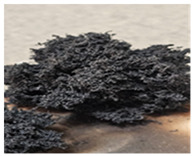	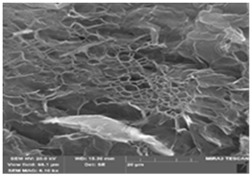	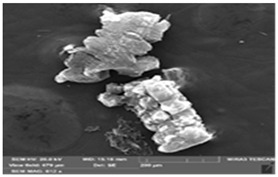
4	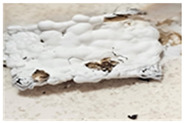	The char layer was extremely brittle; therefore, capturing high-quality photographs was not feasible.	The char layer is extremely brittle; therefore, capturing high-quality photographs was not feasible.
5	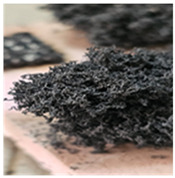	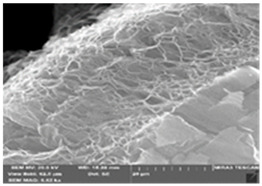	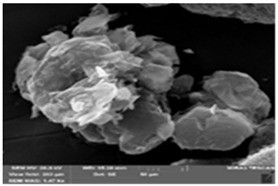
6	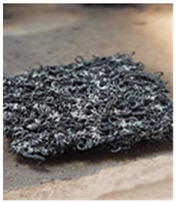	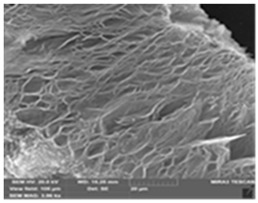	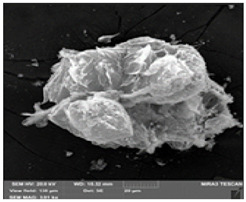

**Table 6:** Summary of thermogravimetric analysis results.

Sample No.	Endothermic Peak (Temperature Range and Absorbed Energy)	Exothermic Peak (Temperature Range and Heat Released)	Temperature T °C at 1% Mass Loss	Total Mass Loss During Test
1 (A)	(1) 220.37–267.91 °C; 6.61 J/g (2) 378.83–474.77 °C; 142.87 J/g	(1) 267.91–335.32 °C; 35.25 J/g; (2) 450.86–525.19 °C 269.62 J/g	191.40 °C	61.006%
2 (S)	(1) 234.18–296.30 °C 14.74 J/g (2) 297.16–389.35 °C 84.11 J/g	358.43–491.85 °C; 197.90 J/g	218.46 °C	29.069%
3 (S)	236.87–286.54 °C 21.92 J/g	356.21–462.13 °C; 195.93 J/g	53.26 °C	26.172%
4 (S)	-	(1) 334.45–446.11 °C; 203.26 J/g (2) 459.06–477.48 °C; 2.96 J/g (3) 490.15–569.86 °C; 227.62 J/g	164.75 °C	55.569%
5 (A)	213.47–276.77 °C 28.30 J/g	(1) 234.48–418.62 °C; 325.08 J/g (2) 440.49–556.15 °C; 163.53 J/g	152.97 °C	60.757%
6 (A)	-	(1) 254.67–427.82 °C; 337.54 J/g (2) 449.39–583.71 °C 563.69 J/g	191.48 °C	74.903%

**Table 7:** Summary of small-scale fire test results.

Sample No.	1	2	3	4	5	6
IG Content	5%	25%	40%	0%	40%	25%
Limit State	20 min	40 min	43 min 30 s	16 min 30 s	44 min 20 s	36 min 40 s
Fire-Protective Efficiency [37]	Group 7	Group 6	Group 6	Group 7	Group 6	Group 6

**Table 8:** Mechanical properties of the specimens.

Sample	Tensile Modulus, *E_p_*, N/mm^2^	Elongation at Break, mm	Max. Stress, N/mm^2^	Elongation at Break, %	Thickness, mm	Width, mm
No. 2 *	85.123/ 48.761	5.36/ 4.52	7.06/ 1.29	5.21/ 4.18	1.45	6.51
No. 3 *	260.23/ 470.28	4.02 4.22	12.01/ 21.78	3.90/ 4.10	1.5	6.55
No. 5 *	158.607 **/ 228.917 ***	7.84/ 4.77	8.35/ 8.17	10.83 /5.86	1.57 *	6.56 *

* Average of 5 specimens; ** Warp direction, “TRANSVERSE” (numerator); *** Weft direction, “LONGITUDINAL” (denominator).

**Table 9:** Summary of results for the primer systems.

Accelerated Weathering Tests		
	Without fire-protective mesh	With fire-protective mesh
Appearance	No changes	No changes
X-cut adhesion	Grade 1	-
Cross-cut adhesion	Grade 1	Grade 1
Salt Spray Chamber Tests		
	Without fire-protective mesh	With fire-protective mesh
Appearance	Corrosion of the product	Corrosion of the product
Cross-cut adhesion	Grade 1	Grade 1

## Data Availability

The original contributions presented in this study are included in the article. Further inquiries can be directed to the corresponding author.
